# Infrequent detection of *Pneumocystis jirovecii *by PCR in oral wash specimens from TB patients with or without HIV and healthy contacts in Tanzania

**DOI:** 10.1186/1471-2334-10-140

**Published:** 2010-05-28

**Authors:** Lotte Jensen, Andreas V Jensen, George Praygod, Jeremiah Kidola, Daniel Faurholt-Jepsen, John Changalucha, Nyagosya Range, Henrik Friis, Jannik Helweg-Larsen, Jorgen S Jensen, Aase B Andersen

**Affiliations:** 1 Department of Infectious Diseases, Rigshospitalet, Denmark; 2 Department of Human Nutrition, University of Copenhagen, Denmark; 3 National Institute for Medical Research, Mwanza, Tanzania; 4 National Institute for Medical Research, Muhimbili Centre, Dar es Salaam, Tanzania; 5 Department of Bacteriology, Mycology, and Parasitology, Statens Serum Institut, Copenhagen, Denmark

## Abstract

**Background:**

In tuberculosis (TB) endemic parts of the world, patients with pulmonary symptoms are managed as "smear-negative TB patients" if they do not improve on a two-week presumptive, broad-spectrum course of antibiotic treatment even if they are TB microscopy smear negative. These patients are frequently HIV positive and have a higher mortality than smear-positive TB patients. Lack of access to diagnose *Pneumocystis jirovecii *pneumonia might be a contributing reason. We therefore assessed the prevalence of *P. jirovecii *by PCR in oral wash specimens among TB patients and healthy individuals in an HIV- and TB-endemic area of sub-Saharan Africa.

**Methods:**

A prospective study of 384 patients initiating treatment for sputum smear-positive and smear-negative TB and 100 healthy household contacts and neighbourhood controls.

DNA from oral wash specimens was examined by PCR for *P. jirovecii*. All patients delivered sputum for TB microscopy and culture. Healthy contacts and community controls were clinically assessed and all study subjects were HIV tested and had CD4 cell counts determined. Clinical status and mortality was assessed after a follow-up period of 5 months.

**Results:**

384 patients and 100 controls were included, 53% and 8% HIV positive respectively. A total number of 65 patients and controls (13.6%) were at definitive risk for PCP based on CD4 counts <200 cells per mm^3 ^and no specific PCP prophylaxis. Only a single patient (0.3% of the patients) was PCR positive for *P. jirovecii*. None of the healthy household contacts or neighbourhood controls had PCR-detectable *P. jirovecii *DNA in their oral wash specimens regardless of HIV-status.

**Conclusions:**

The prevalence of *P. jirovecii *as detected by PCR on oral wash specimens was very low among TB patients with or without HIV and healthy individuals in Tanzania. Colonisation by *P. jirovecii *was not detected among healthy controls. The present findings may encourage diagnostic use of this non-invasive method.

## Background

*Pneumocystis jirovecii *pneumonia (PCP) remains a relatively common and serious opportunistic infection among HIV infected in Western countries, even in the era of antiretroviral therapy (ART) [1,2]. In Africa, PCP is common and often fatal in HIV infected infants less than 1 year of age [3]. Data regarding adult patients from Uganda [4], Malawi [5] and Ethiopia [6] have shown *P. jirovecii *prevalence varying from 9% to 38% among smear-negative, mainly HIV-positive TB patients.

The present study was inspired by results of a study performed in Mwanza, Tanzania, in which we observed a HIV prevalence of 63% among patients with smear-negative TB, according to WHO classification [7]. Among the smear-positive TB patients the HIV prevalence was only 44% but the HIV-positive patients had a mortality of 23% compared to 4% among HIV-negative patients [8]. We considered whether undiagnosed PCP could contribute to the excess mortality in these HIV-positive patients.

Oral wash specimens with subsequent PCR detection of *P. jirovecii *DNA has been reported to be a non-invasive and easy-to-perform procedure with a diagnostic sensitivity up to 89% [9-11]. Because very little is known about the geographical variations in the prevalence of latent *P. jirovecii *colonisation we decided to conduct a study on the applicability of an oral wash procedure in an HIV- and TB-endemic region of sub-Saharan Africa including both healthy community controls, household contacts, and clinically-ill patients suspected of pulmonary TB.

## Methods

### Study environment

The study was conducted in Mwanza City, North-western Tanzania from April 2007 to March 2009. The study was a part of a TB and nutrition study (ClinicalTrials.org, registration number NCT00311298) in which pulmonary TB (PTB) patients were treated according to the national guidelines for TB [12] and randomized to receive micronutrients and energy-protein supplements in different amounts.

The patients were treated according to national guidelines. In Tanzania, HIV testing is offered as part of the routine medical management of TB patients [12] in line with the UNAIDS statement 2004 [13]. The HIV prevalence among incident TB cases is estimated to be 47% (year 2007) [14]. Antiretroviral treatment is available for HIV patients and is prescribed in accordance with WHO guidelines [15,16]. Prophylactic *P. jirovecii *treatment of HIV-positive adults with co-trimoxazole is offered to WHO stage 3 (which includes PTB) patients, to patients with symptomatic HIV disease and to asymptomatic HIV-positive individuals with CD4 count <200 cells per mm^3 ^[15].

### Patient recruitment and eligibility

PTB patients diagnosed at two hospitals and two health centres and about to start treatment under the national TB programme were approached for participation in the study. Exclusion criteria were: age below 15 years, pregnancy, terminal illness (judged unlikely to survive for 48 hours), and not staying in Mwanza for the entire 5 month follow-up period. Patients who did not show up for 5 month follow-up and could not be traced were considered as defaulted.

One household contact and one neighbourhood control was recruited for each sputum smear-positive TB patient. With the acceptance from the PTB patient, staff from the study team visited the house of the PTB patient and chose a participant by lot among the eligible household members. Lot-selected sex- and age-matched neighbourhood controls were identified with the assistance of the local *ten-cell-leaders *among eligible residents of the area.

### Ethical considerations

The national ethics committees in Denmark and Tanzania approved the study. All participants were informed orally in their local tongue (Kiswahili or Sukuma language) and in writing (Kiswahili) before written consent were obtained and they were free to withdraw from the study at any time. Pre-HIV test counselling was provided to the study subjects. All study subjects who were tested for HIV received post-HIV test counselling regardless of the result. If the HIV test was positive the subject was referred to the local HIV programme.

### TB diagnostics

All patients produced three sputum samples, of which at least one was a morning sample, for microscopic examination after Ziehl-Neelsen staining. As part of this study, an extra sample was obtained for control microscopy (Auramine-O staining) and culture on Lowenstein-Jensen solid media at the Zonal Reference Laboratory at Bugando Medical Centre.

PTB positive patients had a positive culture and/or microscopy positive result. PTB negative patients had a negative culture and were found negative by microscopy but were considered eligible for TB treatment on clinical suspicion and chest X-ray, often after lack of improvement following two weeks of presumptive broad spectrum antibiotic treatment. The choices of antibiotics varied and a presumptive curative treatment was expected to cure bacterial pneumonia.

### HIV testing and CD4 counts

HIV testing was performed using Determine HIV 1/2 (Inverness Medical Innovations, Inc., Delaware, USA) and Capillus HIV-1/HIV-2 (Trinity Biotech Plc., Wicklow, Ireland). If discordance was found between the two tests a confirmatory ELISA test was performed (Organon Uniform II, Organon Teknia, NL). CD4 cell count was determined using Partec Cyflow counter (Partec, GmbH, Münster, FRG).

### Oral wash specimens

All patients enrolled in the study had the oral wash procedure performed within the first week of diagnosis and treatment initiation of TB. The controls had the oral wash procedure performed at the same day as all other measurements were taken. The participants were fasting since midnight and were asked to postpone brushing their teeth until after the oral wash, which included rinsing/gargling the mouth with 10 ml of sterile saline for 1 min. The procedure was monitored by a staff member. The samples were collected in sterile tubes and centrifuged at 3000 × g for 30 min. One ml of sediment was stored frozen at -80°C until transfer to Denmark on dry ice.

### Detection of *P. jirovecii *from oral wash specimens

DNA was extracted from oral wash specimens by Chelex extraction and 2 μl of the supernatant was used for touch-down-PCR detecting a fragment of the *P. jirovecii *mitochondrial large subunit rRNA gene as previously described [10,11]. An internal process control was included for detecting the presence of *Taq *DNA polymerase inhibitors or suboptimal reaction conditions. Positive results were confirmed by two different PCRs amplifying mitochondrial small subunit rRNA and major surface glycoprotein of *P. jirovecii *[17], respectively.

A specimen was regarded as positive for *P. jirovecii *when at least two of the tests were positive. If inhibition was observed, the test was repeated with 1 μl of the sample as template.

In order to validate the presence of human DNA in the samples an additional PCR was performed for the detection of a 307 bp fragment of human betaglobin.

### Statistical analysis

Data were collected daily onto data collection forms, checked for accuracy and entered into EpiData version 3.1 (The EpiData Association, Odense, Denmark, 2008). Data were exported to STATA 10.1 (StataCorp, College Station, Texas, USA, 2009) for statistical analysis. Fishers exact test was used to assess significant associations among categorical variables. For numerical data Wilcoxon-Mann-Whitney test was used.

## Results

**Figure 1:**
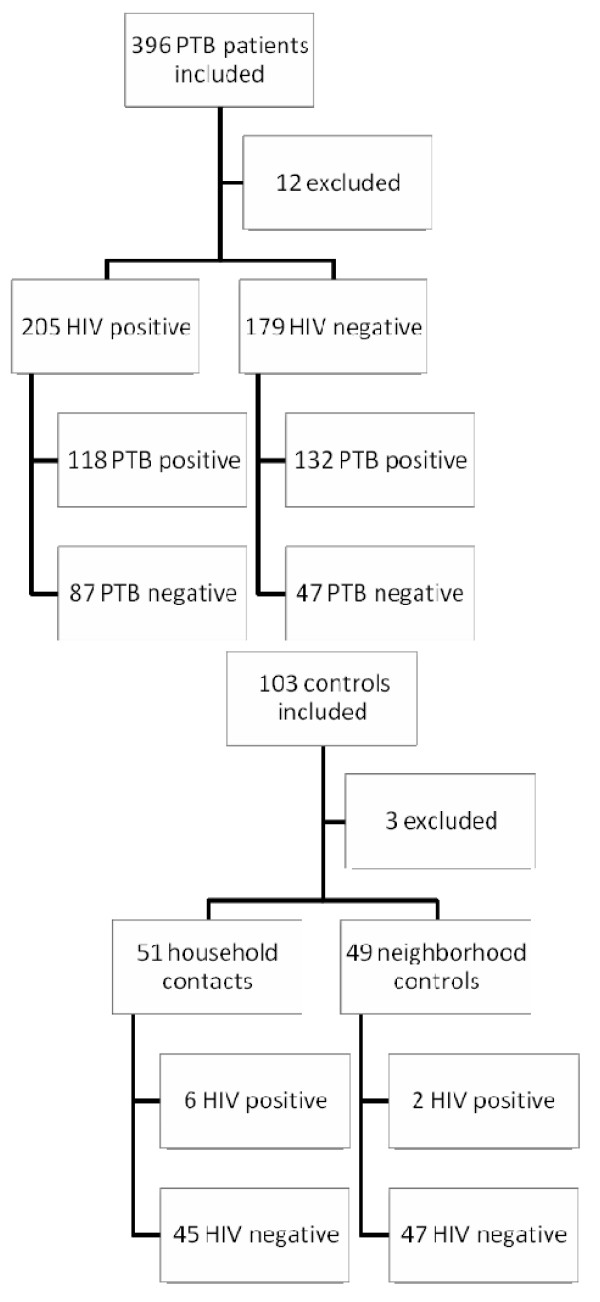
**Study recruitment and flow chart**. PTB patients: 11 were excluded because betaglobin PCR was negative (7 HIV positive, 4 HIV negative) and 1 because HIV status was missing. Controls: 3 were excluded because betaglobin PCR was negative (1 household contact and 2 neighbourhood controls).

Totals of 396 PTB patients and 103 healthy controls were enrolled. Twelve patients were excluded: One because of lacking HIV result, 11 because of poor quality sample material containing no detectable betaglobin DNA (7 HIV positive, 4 HIV negative). Three controls were excluded because of poor sample material (one household contact and two neighbourhood controls) (Flow chart depicted in Figure 1). The characteristics of the participants are shown in Table 1. The median age was 33 years (range 15 - 89 years). The HIV-positive and HIV-negative patients had similar median age of 34 versus 32 years (p = 0.16) and did not differ from the control group with a median age of 32 years (*p *= 0.08). The gender distribution was even with 220 (45.5%) of the study subjects being female. Mortality and loss to follow up was assessed after five months.

**Table 1:** Characteristics of study subjects

Characteristics	All	HIV positive patients	HIV negative patients	Household contacts	Neighborhood controls
Number of study subjects	484	205	179	51	49
Female (%)	220 (45.5)	99 (48.3)	64 (36.0)	33 (64.7)	24 (49.0)
Age median (range)	33 (15-89)	34 (19-70)	32 (15-89)	32 (18-86)	33 (16-55)
Males	36 (15-89)	37 (23-70)	35 (15-89)	36 (18-60)	32 (16-55)
Females	32 (16-86)	32 (19-60)	30 (18-77)	30 (18-86)	33 (16-55)
HIV positive (%)	213 (44.0)	205 (100)	0	6 (11.8)	2 (4.1)
TB positive (%)	250 (65.1)	118 (57.6)	132 (73.7)	-	-
CD4* median (range)	340 (21-2117)	208 (21-926)	387 (49-1609)	622 (146-1714)	465 (180-2117)
CD4* < 200 (%)	112 (23.1)	94 (45.9)	15 (8.4)	1 (2.0)	2 (4.1)
Receiving presumptive antibiotic treatment (%)	175 (46.5)	98 (49.0)	77 (43.8)	-	-
Receiving presumptive curative TMX**(%)	45 (12.3)	35 (17.8)	10 (5.9)	-	-
Receiving prophylactic TMX**^1 ^(%)	57 (57.6)	57 (57.6)	-	-	-

All: Information on sex was missing for 1 patient, age was missing for 1 patient, information on presumptive antibiotic treatment was missing for 8 patients, information on presumptive curative TMX missing for 10 patients.

HIV positive: information on presumptive antibiotic treatment was missing for 5 patients. Information on presumptive curative TMX was missing for 3 patients.

HIV negative: age missing for 1 patient, sex missing for 1 patient. Information regarding presumptive antibiotic treatment was missing for 3 patients, information on presumptive curative TMX missing for 7 patients.

*CD4, number of CD4 cells per mm3. **TMX: cotrimoxazole

^1 ^Number of patients receiving prophylactic TMX out of all patients known HIV positive before study entry.

### HIV and PTB status

A total of 205 (53.4%) patients and 8 (8%) controls were found to be HIV positive. 118 (57.6%) of the HIV-positive suspected TB patients were diagnosed with smear-positive TB i.e. PTB positive. The median CD4 count for the HIV-positive patients was 208 cells per mm^3^, which was significantly lower than the median count of 387 cells per mm^3 ^found in the HIV-negative group (*p *< 0.01) and 538 cells per mm^3 ^in the control group (*p *< 0.01).

CD4 count <200 cells per mm^3 ^was noted in 112 (23%) of all the participants, of which 94 (84%) were HIV positive. Fifteen (8%) of the HIV-negative patients and 3 (3%) from the control group also had CD4 counts below 200 cells per mm^3^. The median CD4 count did not differ in the HIV-positive, PTB negative group (219 cells per mm^3^) compared to the HIV-positive, PTB positive group (203 cells per mm^3^) (*p *= 0.89).

### PCP detection

**Figure 2:**
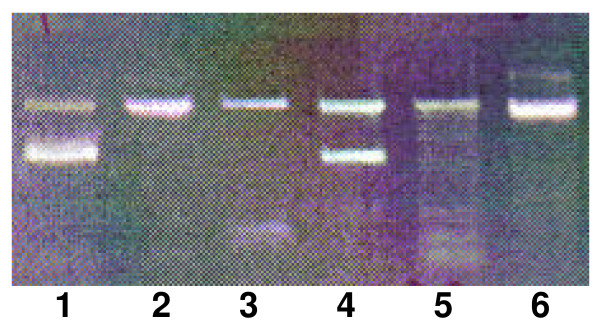
**PCR products subjected to agarose gel electrophoresis**. **Lane 1**: a positive control with amplified DNA from purified *P. jirovecii *from lung tissue. **Lanes 2, 3, 5 and 6 **are negative samples amplifying the internal process control. **Lane 4**: Sample from patient. *P. jirovecii *reaction is positive as both the internal process control and *P. jirovecii *DNA of 346 base pairs are amplified.

Only a single patient (0.3%) tested positive with PCR for *P. jirovecii *(Figure 2). This 37-year-old male patient was HIV positive, PTB positive with CD4 count of 85 cells per mm^3^. The patient was not known to be HIV positive before study entry and had not received cotrimoxazole treatment before study entry. The patient died about one month after study entry at one of the hospitals where the study took place. None of the healthy household contacts or neighbourhood controls tested positive for *P. jirovecii*.

### Presumptive antibiotic treatment

None of the household contacts or neighbourhood controls had received antibiotics within two weeks before enrolling the study. In contrast 175 (47%) of the TB suspects had received one or more presumptive curative courses of antibiotic treatments for two weeks before enrolling the study. The most commonly used antibiotics were amoxycillin, erythromycin and cotrimoxazole. There was no difference between the pre-treatment frequencies of the HIV-positive versus the HIV-negative group (*p *= 0.35). 18% (35, data for 8 missing) of the HIV-positive patients had received cotrimoxazole as opposed to 5.9% (10, data for 10 missing) of HIV-negative patients (*p *< 0.01).

### Prophylactic cotrimoxazole

Prophylactic cotrimoxazole was used by 57 (58%) of the patients, known to be HIV positive before study entry with a median of 60 days (range 2-1095) (data missing for 12 patients). Among study subjects at risk with a CD4 cell count <200 cells per mm^3^, 75 (70.1%) had not received prophylaxis (data missing for 5 persons). Eight (53.3%) of the HIV-positive patients, who died, knew their HIV status at study entry. Four of them had received prophylaxis, three of them for a median of 30 days (range 21-330) (information on duration missing for one patient).

65 study participants (62 patients and 3 controls) had a CD4 count <200 cells per mm^3 ^and had neither received prophylactic nor curative treatment with cotrimoxazole and were therefore susceptible to *P. jirovecii *infection (data missing for 5 persons).

### Dead or lost to follow up

Twenty patients (5.6%) had died at 5 months follow-up, 12 patients defaulted and 12 patients had moved away from the region. Significantly more patients died in the HIV-positive group, 15 (7.8%) than in the HIV-negative group, 5 (3.0%) (*p *= 0.04).

No significant difference was found in the number of patients who died between the HIV-positive, PTB negative group, 7 (8.8%) and the HIV-positive, PTB positive group, 8 (7.1%) (*p *= 0.43).

## Discussion

Recent studies have indicated that the incidence of symptomatic PCP may have been underreported in sub-Saharan Africa. *P. jirovecii *was diagnosed in bronchoalveolar lavage (BAL) fluid from 9% of smear-negative, mainly HIV-positive, TB patients in Malawi [5]. In similar studies from Ethiopia and Uganda, PCP was the definitive diagnosis in one third of HIV-positive, PTB smear-negative patients, who underwent BAL [4,6]. In a community based Malawian study PCP was diagnosed in only 1 per 100 person years of follow-up, but strongly correlated to HIV status and increasing with declining CD4 count [18]. The differences in prevalence reported from different studies may to some extent be explained by the patients included and selection bias introduced by e.g. including hospitalised patients versus patient referred to out patient clinics, like in our study.

Obviously, the technical requirements and resources needed for bronchoscopy-based procedures prevent BAL as a routine option in low-income settings. Induced sputum procedures are aerosol inducing and difficult to handle safely especially when applied to patients suspected of TB. A diagnostic method based on oral wash specimens is a non-invasive, easily-obtained procedure but the sensitive PCR entails the risk of also detecting DNA in persons only colonised with *P. jirovecii*. Reported carrier frequencies vary in different populations using different PCR methods. A study from Spain found *Pneumocystis *DNA in 20% of oral wash specimens from 50 healthy - but hospital-affiliated persons [19]. A small study of 44 HIV-infected adults from the USA detected colonization in 11.4% using a nested PCR method [20]. *P. jirovecii *DNA was detected in 18% of BAL fluids from HIV-negative pulmonary patients undergoing bronchoscopy for other reasons especially if treated with prednisolone [21]. A study of autopsy samples from American HIV patients, who had died from other reasons than PCP had *P. jirovecii *DNA detectable by a sensitive, nested PCR method in 42 of 91 subjects [22].

In this study only one of 384 patients suspected of TB tested positive for *P. jirovecii*. The patient was HIV positive with a CD4 count of 83 per mm^3^, no prior prophylactic or curative treatment attempts with cotrimoxazole, and the clinical course resulting in death after two months of TB treatment supports the diagnosis. The low infection rate may be ascribed to a higher PCP prophylaxis coverage in this study compared to the Ethiopian, Ugandan and Malawian studies [4-6]. In this study, 58% of the known HIV positive patients at risk actually received prophylaxis. However, a large group of 65 patients and controls (13.6% of the total study population) fulfilling the criteria for prophylaxis had not received prophylaxis or presumptive cotrimoxazole curative treatment and were therefore susceptible to *P. jirovecii *infection. Recalculating the PCP prevalence for the TB patient subgroup results in a rate of 1.6% (CI: 0-9.8), which is overlapping the rates found in the Malawian study by Hargreaves et al. [5]. Twenty patients died during the five months of observation and 24 were lost to follow up. The mortality was higher in the HIV-positive than in the HIV-negative group (*p *= 0.04), but in this study only part of the excess mortality could be explained by PCP.

None of the 100 healthy household contacts and neighbourhood controls tested positive for *P. jirovecii*. The carrier frequency seems to be low or at least below the detection limit of the PCR method applied in this study. The sensitivity of the *P. jirovecii *specific PCR used in this study has in other settings been shown to detect 89% of PCPs using Giemsa and immunofluorescence staining of bronchoalveolar fluid as reference material [10]. It might be possible to increase the diagnostic sensitivity by asking the patients to cough before the oral wash to mobilise material from lower parts of the respiratory tract. However, a PCR detecting a segment of the human betaglobin gene was performed in order to assure the presence of amplifiable DNA fragments and thereby assess the quality of the sample material. The length of the betaglobin PCR product was similar to the *P. jirovecii *target. Only 14 participants were excluded from the study due to absence of amplifiable DNA in the samples, suggesting that the performance of the present study is similar to other studies in which the same PCR method was used. Colonisation may occur transiently and therefore some studies have examined repeated samples obtained at different time points [19]. The relatively high number of study participants to some extent counteracts this problem.

## Conclusion

This study did not support the hypothesis that colonization with *P. jirovecii *is widespread, at least not in this sub-Saharan region although larger studies would be needed to support this. If confirmed, this may increase the applicability of the very easy-to-perform oral wash procedure as a supplement in the diagnosis of the otherwise lethal PCP in HIV-positive patients with respiratory symptoms.

## Competing interests

The authors declare that they have no competing interests.

## Authors' contributions

LJ and AVJ conducted the oral wash sampling and processing. LJ, JHL, and JSJ performed the PCR analyses. GP, JK, DFJ, JC, HF, NR, and ABA conducted the background study and designed the present study. LJ prepared the first draft of this manuscript and all authors read and approved the final manuscript.

## Pre-publication history

The pre-publication history for this paper can be accessed here:

http://www.biomedcentral.com/1471-2334/10/140/prepub
